# Study of the Cytotoxic Effects of the New Synthetic Isothiocyanate CM9 and Its Fullerene Derivative on Human T-Leukemia Cells

**DOI:** 10.3390/toxins7020535

**Published:** 2015-02-11

**Authors:** Elena De Gianni, Eleonora Turrini, Andrea Milelli, Francesca Maffei, Marco Carini, Anna Minarini, Vincenzo Tumiatti, Tatiana Da Ros, Maurizio Prato, Carmela Fimognari

**Affiliations:** 1 Interdepartmental Centre for Industrial Research in Advanced Mechanical Engineering Applications and Materials Technology, *Alma Mater Studiorum*-University of Bologna, Piazza Malatesta, 29/30, 47923 Rimini, Italy; E-Mail: elena.degianni2@unibo.it; 2 Department for Life Quality Studies, *Alma Mater Studiorum*-University of Bologna, Corso d’Augusto 237, 47921 Rimini, Italy; E-Mails: eleonora.turrini@unibo.it (E.T.); andrea.milelli3@unibo.it (A.M.); francesca.maffei@unibo.it (F.M.); vincenzo.tumiatti@unibo.it (V.T.); 3 Department of Chemical and Pharmaceutical Sciences, University of Trieste, Piazzale Europa 1, 34127 Trieste, Italy; E-Mails: marco_carini@yahoo.it (M.C.); daros@units.it (T.D.R.); prato@units.it (M.P.); 4 Department of Pharmacy and Biotechnology, *Alma Mater Studiorum*-University of Bologna, via Belmeloro 6, 40126 Bologna, Italy; E-Mail: anna.minarini@unibo.it

**Keywords:** fullerene, isothiocyanate, naphthalene diimide, cancer, apoptosis, cell proliferation, p53, doxorubicin, genotoxicity

## Abstract

One important strategy to develop effective anticancer agents is based on natural products. Many active phytochemicals are in human clinical trials and have been used for a long time, alone and in association with conventional anticancer drugs, for the treatment of various types of cancers. A great number of *in vitro*, *in vivo* and clinical reports document the multi-target anticancer activities of isothiocyanates and of compounds characterized by a naphthalenetetracarboxylic diimide scaffold. In order to search for new anticancer agents with a better pharmaco-toxicological profile, we investigated hybrid compounds obtained by inserting isothiocyanate group(s) on a naphthalenetetracarboxylic diimide scaffold. Moreover, since water-soluble fullerene derivatives can cross cell membranes thus favoring the delivery of anticancer therapeutics, we explored the cytostatic and cytotoxic activity of hybrid compounds conjugated with fullerene. We studied their cytostatic and cytotoxic effects on a human T-lymphoblastoid cell line by using different flow cytometric assays. In order to better understand their pharmaco-toxicological potential, we also analyzed their genotoxicity. Our global results show that the synthesized compounds reduced significantly the viability of leukemia cells. However, the conjugation with a non-toxic vector did not increase their anticancer potential. This opens an interesting research pattern for certain fullerene properties.

## 1. Introduction

Increasing recurrence of tumors and severe side-effects of chemotherapeutic agents represent the main causes of the reduced clinical efficacy of several anticancer agents that are currently used. Thus, there is a constant need for more effective and less toxic anticancer therapies. One important strategy to develop effective anticancer agents is based on natural products. Many active phytochemicals are in human clinical trials and have been used for a long time, alone and in association with conventional anticancer drugs, for the treatment of various types of cancers.

A great number of *in vitro*, *in vivo* and clinical reports document the anticancer activities of isothiocyanates (ITCs). They are effective in the prevention and treatment of different cancer types [1]. In particular, they are able to block cell proliferation, induce apoptosis [2], interfere with all essential steps of neovascularization [3], and inhibit the metastatic potential of cancer cells [4]. Moreover, some studies reported the ability of ITCs to increase the anticancer efficacy of conventional anticancer drugs [5,6].

Compounds characterized by a naphthalenetetracarboxylic diimide (NDI) scaffold exhibit *bis*-threading intercalating ability [7], enhance the stabilization of DNA triplexes [8], and stabilize [9] or alkylate [10] G-quadruplex DNA structure [11,12]. Moreover, an NDI derivative (i) is able to strongly stabilize G-quadruplex sequences located both at telomeric ends and at the promoter region of oncogenes like c-KIT; (ii) it induces a suppression of KIT mRNA and protein expression in patient-derived gastrointestinal stromal tumors, thus representing an alternative promising approach for the treatment of human gastrointestinal stromal cancer [13].

In order to search new anticancer agents with a better pharmaco-toxicological profile, we investigated hybrid compounds obtained by inserting ITC group(s) on an NDI scaffold [14].

Some years ago, the National Cancer Institute recognized nanotechnology as a paradigm-changing opportunity thanks to its potential to enable significant breakthroughs in cancer therapy [15]. Accordingly, the research on C_60_ has become a topic of considerable interest in medicinal and pharmaceutical research due to its unique geometrical shapes, as well as novel photophysical properties. Fullerenes can be loaded with one or more payloads such as chemotherapeutics. In order to overcome the inherent hydrophobicity of the C_60_ sphere, water-soluble fullerene-based derivatives, especially fullerene-containing biomolecules, have been widely explored [16]. The discovery that water-soluble fullerene derivatives can cross cell membranes [17] has raised great interest in biological applications of fullerenes as carriers of chemotherapeutics. However, the application of fullerenes as carriers of chemotherapeutics is still in a very initial phase as there are only a few reports exploring fullerene derivatives as sources of delivery for anticancer therapeutics [18,19,20,21].

**Figure 1:**
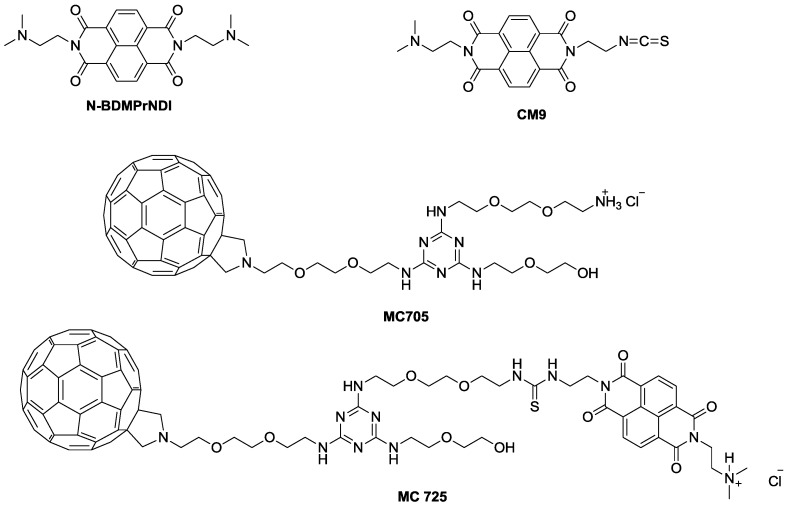
Chemical structure of N-BDMPrNDI, CM9, MC705 and MC725.

In the present study, we explored the molecular events modulating the previously reported cytotoxic activity of CM9 [14] and its effects on cell viability when administered together with doxorubicin. CM9 is an asymmetric NDI constituted of two different side chains: One bearing a protonated dimethylaminoethyl side chain deriving from *N*,*N*'-*bis*[3,3'-(dimethylamino)propylamine]-naphthalene-1,4,5,8-tetracarboxylic diimide (N-BDMPrNDI), and the second one characterized by the ITC group (Figure 1). Furthermore, we conjugated CM9 with a water-soluble fullerene derivative (MC705) (Figure 1) and explored the *in vitro* anticancer potential of the obtained CM9-fullerene derivative (MC725) (Figure 1) through the analysis of its cytostatic and cytotoxic effects on a human T-lymphoblastoid cell line and a human lymphoma cell line. To better understand the pharmaco-toxicological potential of MC725, we also analyzed its genotoxicity.

The pharmacological and genotoxic effects of MC725 were compared with those of MC705, CM9 and the NDI derivative (N-BDMPrNDI) (Figure 1).

## 2. Results and Discussion

This study aimed to identify the molecular mechanisms responsible for the cytotoxic effectiveness of a new synthetic ITC mounted on an NDI scaffold against human Jurkat acute lymphoid leukemia cells and to investigate the *in vitro* anticancer effects of its fullerene conjugate.

**Figure 2:**
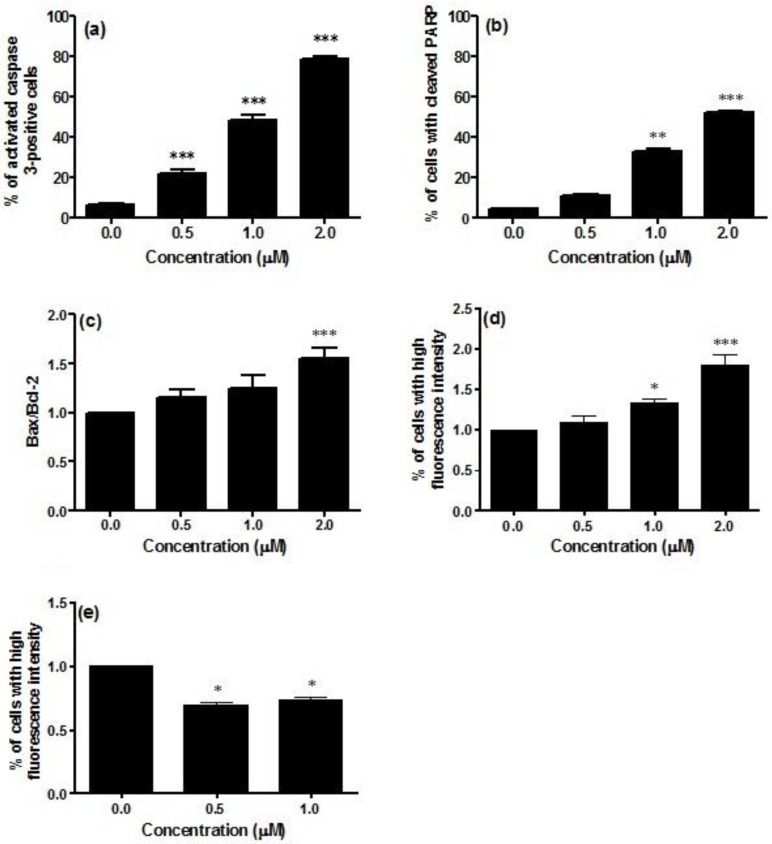
Analysis of caspase-3 activation (**a**); cleavage of PARP (poly ADP ribose polymerase) (**b**); Bax-to-Bcl-2 ratio (**c**); p53 (**d**); and cyclin E (**e**) protein levels after 24 h treatment of Jurkat cells with CM9. After treatment of cells with the indicated CM9 concentrations, antibodies against cleaved-caspase-3, PARP, Bax, Bcl-2, p53, and cyclin E were used, and proteins expression was determined by flow cytometry. All results are expressed as mean ± SEM of at least three experiments. * *p* < 0.05, ** *p* < 0.01, *** *p* < 0.001 *vs.* control.

Caspase-3 activity was significantly increased in Jurkat cells treated with CM9. The percentage of activated caspase-3 cells in non-treated cultures was about 6.3%, which was increased to 78.5% in cells treated with CM9 at 2.0 μM concentration (Figure 2a). An important reporter for caspase-3 activation is PARP (poly ADP ribose polymerase). CM9 induced PARP cleavage at all tested concentrations. After labeling with FITC 85 kDa fragment of cleaved PARP, a five-fold increase in the fraction of cells with cleaved PARP was observed at 2.0 μM (53.0% *vs.* 11.2%), thus confirming caspase-3 activation following CM9 treatment (Figure 2b).

Bax, Bcl-2, and p53 play a critical role in the regulation of apoptosis. In particular, Bax and Bcl-2 are involved in the intrinsic or mitochondrial apoptotic pathway. We have previously reported that CM9 caused a strong drop in ΔΨm. That was demonstrated by a number of cells with decreased mitochondrial potential of about 100% [14].

The stimulation of the intrinsic pathway is characterized by changes in the inner mitochondrial membrane, the opening of the mitochondrial permeability transition pore, the loss of the mitochondrial transmembrane potential, and the release of pro-apoptotic proteins from the mitochondria into the cytosol. Proteins of the Bcl-2 family regulate those apoptotic mitochondrial events [22]. The Bcl-2 family is constituted of proteins with opposing functions, including Bcl-2, which has an anti-apoptotic effect, and Bax with a pro-apoptotic effect [23]. This led us to the investigation of the effect of CM9 on the expression of Bcl-2 and Bax proteins. The evaluation of pro-apoptotic Bax expression revealed that treatment of Jurkat cells with CM9 induced a decrease in Bax expression. In particular, at treatment concentration of 0.5 μM, Bax expression was slightly decreased (0.8% compared to 1.0% in the control). CM9 caused a stronger decrease in Bcl-2 expression (0.6% *vs.* 1.0% in the untreated cultures). Furthermore, it is also possible to observe an increase in the ratio Bax/Bcl-2 at the different concentrations analyzed in Figure 2c. It is interesting to note that different studies showed that overexpression of Bcl-2 protein is a poor prognostic factor in patients with acute leukemia [24,25], and that the change in the Bax/Bcl-2 ratio predisposes to apoptosis cell death [26]. Data presented herein lend further support to this finding, because the treatment with CM9 induced a reduction in the expression of anti-apoptotic Bcl-2 protein, an increase in the Bax/Bcl-2 ratio expression, and induced apoptosis.

P53 activation controls cell fate outcomes, including apoptosis and cell cycle arrest [27], through its binding to multiple binding sites [28]. Along this line, we demonstrated that the expression of p53 was significantly up-regulated in CM9-treated Jurkat cells, with a 1.8-fold increased expression at 2.0 μM compared to untreated cells (Figure 2d). On the whole, taking into account that CM9 increases the Bax/Bcl-2 ratio expression and the expression of p53, our results support the hypothesis that apoptosis only occurs when a certain threshold of transcriptional activation of genes involved in cell apoptosis is reached [27].

As CM9 was previously found to inhibit cell proliferation mediated by an accumulation of cells in the G1 phase and a parallel decrease in the fraction of cells in the S phase [14], we evaluated whether CM9 changes cyclin E level of Jurkat cells.

The cell cycle is regulated by several proteins and it is known that cyclin E is a key transition protein between G1 and S cell cycle phases. A complex balance between its timed synthesis and rapid degradation by the ubiquitin-proteasome system allows the maintenance of the oscillating level of cyclin E during the cell cycle and therefore the unidirectional transition of cells through the G1-S checkpoint [29]. Treatment with CM9 0.5 and 1.0 μM induced 30.8% and 26.4% decrease respectively in cyclin E expression with regard to control (Figure 2e). The modulation of cyclin E by CM9 can be dependent on its ability to attenuate metabolic functions and increase p53 expression. Effectively, a previous study linked attenuated mitochondrial function to cyclin E degradation by p53-induced activation of the ubiquitin-proteasome system [30]. More in-depth molecular studies should be performed to characterize those effects.

**Figure 3:**
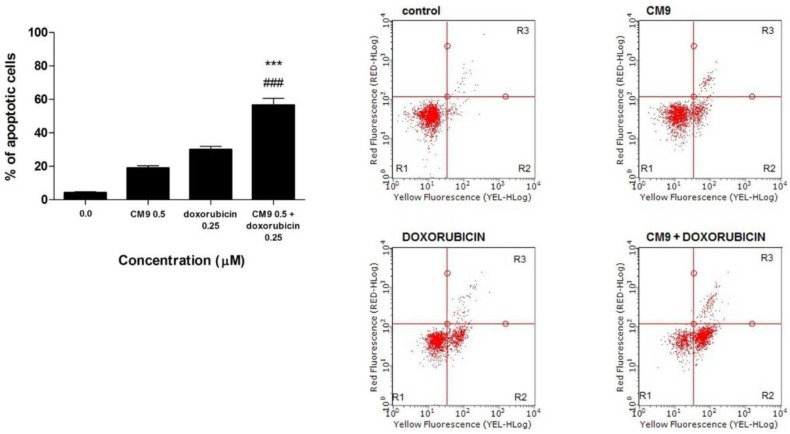
(**a**) Induction of apoptosis after 24 h treatment of Jurkat cells with CM9 (0.5 μM) plus doxorubicin (0.25 μM) in Jurkat cells. Cells were stained with Annexin V/7-amino-actinomycin D (7-AAD), and apoptosis was determined using flow cytometry as described in Materials and Methods. All results are expressed as mean ± SEM of at least three experiments. ### *p* < 0.001 *vs.* doxorubicin; *** *p* < 0.001 *vs.* CM9; (**b**) Representative dot plots of Jurkat cultures untreated and treated with CM9, doxorubicin or CM9 plus doxorubicin for 24 h. Cluster R1: Living cells; cluster R2: Apoptotic cells; cluster R3: Necrotic cells. Percentages of labeled cells as defined by quadrant markers.

A clinically relevant observation is that CM9 increases the cytotoxic efficacy of doxorubicin. Doxorubicin is an anticancer drug belonging to the anthracycline family, the activity of which has been well-documented against liquid and solid tumors. Several studies have actually reported that doxorubicin can induce apoptosis *in vivo* and *in vitro* in many tumor cell types [31]. The co-administration of doxorubicin and CM9 increases the apoptotic effect of the chemotherapeutic drug on Jurkat cells (Figure 3a,b). In particular, the combination of CM9 0.5 μM plus doxorubicin 0.25 μM leads to 56.7% of apoptotic events, compared to 30.0% for doxorubicin or 19.0% for CM9, when cells were treated with the two drugs separately (Figure 3a). The CI was found to be <1, thus showing a synergistic effect between doxorubicin and CM9. Our results show that CM9 can increase the toxic action of doxorubicin by inducing a greater apoptotic tendency of Jurkat leukemic cells.

Taken together, our results demonstrate that CM9 exhibits a marked antitumor effect by inhibiting cell proliferation and inducing apoptosis in the same way. On these bases, we decided to conjugate CM9 with MC705, a water-soluble fullerene, and explored the *in vitro* anticancer potential of the obtained fullerene derivative (MC725) through the analysis of its cytostatic and cytotoxic effects.

**Figure 4:**
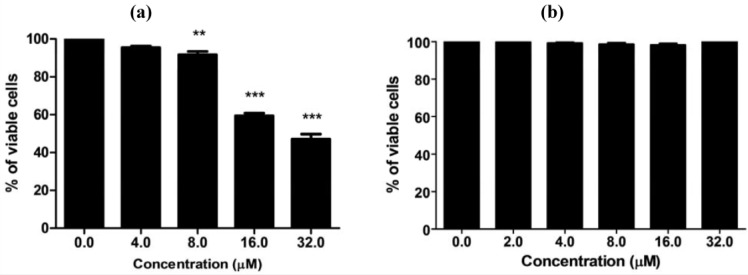
Cell viability after 24 h treatment of Jurkat cells with MC725 (**a**) or MC705 (**b**). Cells were stained with 7-amino-actinomycin D (7-AAD), and viability was determined by flow cytometry. All results are expressed as mean ± SEM of at least three experiments. ** *p* < 0.01, *** *p* < 0.001 *vs.* control.

MC725 significantly decreased Jurkat cell viability starting from 8.0 μM concentration (91.6% of viable cells). Cell viability decreased in a concentration-dependent manner until it was tested at the highest concentration (44.2% at 32.0 μM) (Figure 4a). On the basis of those results, an IC_50_ of 23.8 μM was calculated.

**Figure 5:**
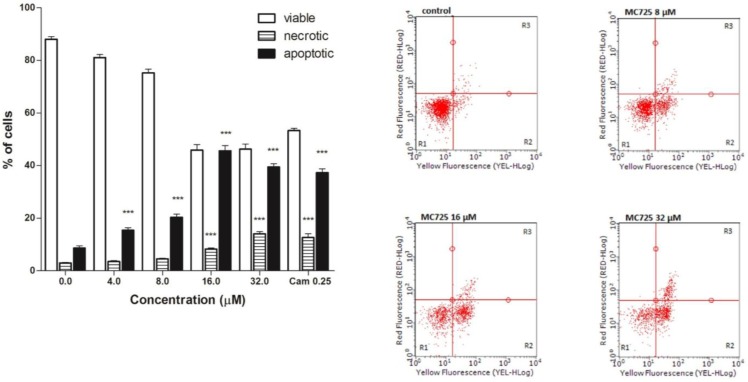
(**a**) Fraction of viable, necrotic and apoptotic cells after 24 h treatment of Jurkat cells with MC725. Cam: camptothecin. Cells were stained with Annexin V/7-amino-actinomycin D (7-AAD), and apoptosis was determined using flow cytometry as described in Materials and Methods. All results are expressed as mean ± SEM of at least three experiments. *** *p* < 0.001 *vs.* control. (**b**) Representative dot plots of Jurkat cultures untreated and treated with MC725 at the indicated concentrations for 24 h. Cluster R1: Living cells; cluster R2: Apoptotic cells; cluster R3: Necrotic cells. Percentages of labeled cells as defined by quadrant markers.

A proapoptotic effect was observed at all tested concentrations for MC725 (Figure 5a,b). The highest effect was recorded at 16.0 μM (47.4% *vs.* 7.7% in the untreated cultures), with a slight induction of necrotic events (Figure 5).

MC705 did not affect cells viability at any tested concentrations (Figure 4b).

**Figure 6:**
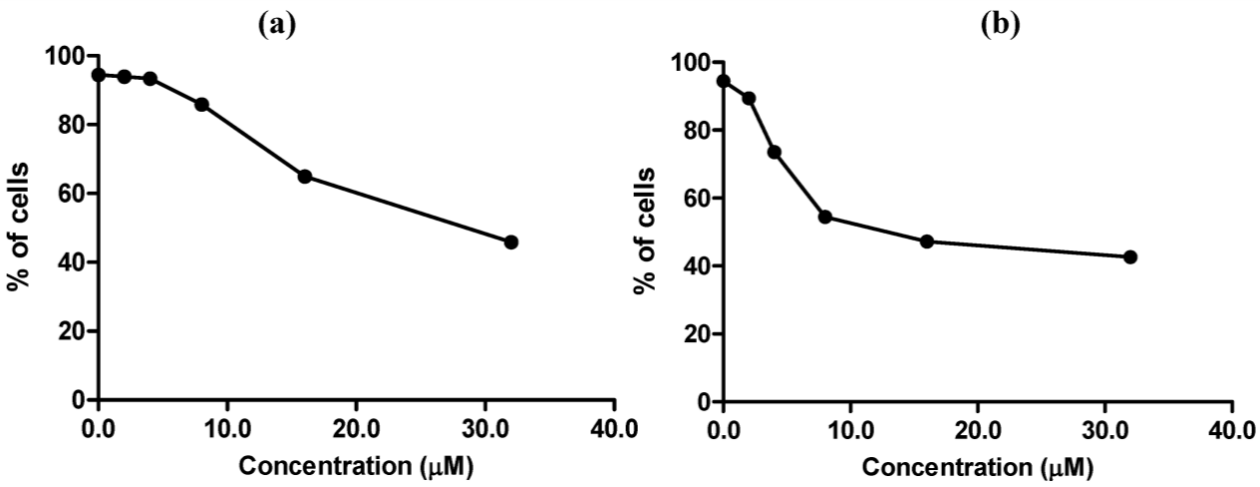
Cell viability after 24 h treatment of Raji cells with MC725 (**a**) or MC705 (**b**). Cells were stained with 7-amino-actinomycin D (7-AAD) and viability was determined by flow cytometry. All results are expressed as mean ± SEM of at least three experiments.

The effects of MC705 and MC725 on cell viability of Raji cells are reported in Figure 6. MC725 had a cytotoxic effect similar to that observed on Jurkat cells (Figure 6a), whereas MC705 evoked a stronger cytotoxic effect than that recorded on Jurkat cells (Figure 6b). The mechanism of cell death was further detailed by the Annexin V/7-AAD, which revealed that the predominant mechanism of cell death induced by MC705 at all tested concentrations was necrosis (data not shown). For the subsequent experiments aimed at investigating the antiproliferative and genotoxic potential of MC705 and MC725, we used Jurkat cells, where we identified a range of concentrations characterized by the presence of an adequate fraction of living cells.

**Figure 7:**
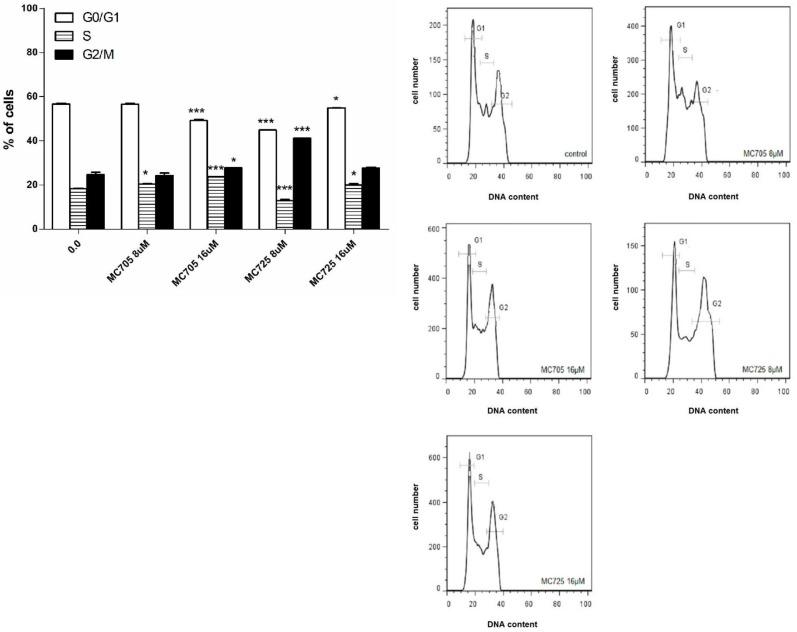
(**a**) Cell-cycle distribution following 24 h treatment of Jurkat cells with MC705 or MC725. Cells were stained with propidium iodide and DNA content was determined by flow cytometry. All results are expressed as mean ± SEM of at least three experiments. * *p* < 0.05, *** *p* < 0.001 *vs.* control; (**b**) Representative flow cytometric histograms following 24 h treatment of Jurkat cells with MC705 or MC725 at the indicated concentrations. Propidium iodide fluorescence of the measured cells is displayed against their number.

The effects of compounds MC725 and MC705 on the cell-cycle progression of Jurkat cells after 24 h of incubation were studied by measuring DNA content through flow cytometry (Figure 7b). We assessed the % of cells in the different phases of cell cycle by setting adjacent cursors without deconvolution of overlapping G0/G1, S, and G2/M phases. MC725 8.0 μM induced an increase of cells in the G2/M phase cells (41.2% compared to 24.6% in the control). Cells in both G1 and S phase significantly decreased (44.9% *vs.* 56.7% in the control and 13.0% *vs.* 18.3% in the control, respectively). Interestingly, the distribution of cells in the different phases of cell cycle changes at 16.0 μM concentration, where we noticed an increase of cells in the S phase (Figure 7a,b).

MC705 16.0 μM, compared to the control, caused an accumulation of cells in the S phase (23.7% *vs.* 18.3%) and in the G2/M phase (27.8% *vs.* 24.6%), accompanied by a decrease in the G1 phase (49.2% *vs.* 56.7%) (Figure 7a,b).

It is interesting to compare these findings with the results from our previous parallel study (using identical methods) on CM9 and the NDI derivative (N-BDMPrNDI) in Jurkat cells [14]. CM9 and N-BDMPrNDI affected cell viability, but to different extents. For example, the dose required to reach IC_50_ was 1.91 μM for CM9 and 3.43 μM for N-BDMPrNDI [14], respectively, indicating that the unconjugated compounds are much more cytotoxic than the conjugated ones.

Another important difference concerns the phase in which the cell-cycle block resides. For CM9, we observed an increase in the percentage of cells in the G1 phase of cell cycle [14]. In contrast, MC725 in the present study induced a marked block in the G2/M phase of the cell cycle, while the fraction of cells in the G1 phase was decreased.

Finally, the fraction of apoptosis induced by CM9 in Jurkat cells (a 9-fold increase with respect to controls) [14] was about two times greater than that recorded for MC725 (2.5-fold with respect to controls). Remarkably, unlike for MC725, at the highest concentrations, necrosis represents the dominant type of cell death for CM9 [14], whereas the % of necrotic cells shown by the number of cells permeable to a vital dye such as 7-AAD in the present study for MC725 was significantly lower than that of apoptotic cells at all the concentrations studied (Figure 5).

Taking into account that the conjugation of CM9 with MC705 was realized by using the ITC group of CM9, it can be useful to compare the bioactivity of MC725 with that of N-BDMPrNDI. N-BDMPrNDI strongly increased the % of apoptotic cells, with the highest apoptotic effect observed at 2 μM (50.0% *vs.* 14.0% of untreated cells). Moreover, N-BDMPrNDI 1.0 μM induced an accumulation of cells in the G1 phase (68.6% *vs.* 56.7% in the control) and a moderate decrease of cells in S phase (13.7% *vs.* 18.3% in the control) and G2/M phase (18.1% *vs.* 24.6% in the control).

**Figure 8:**
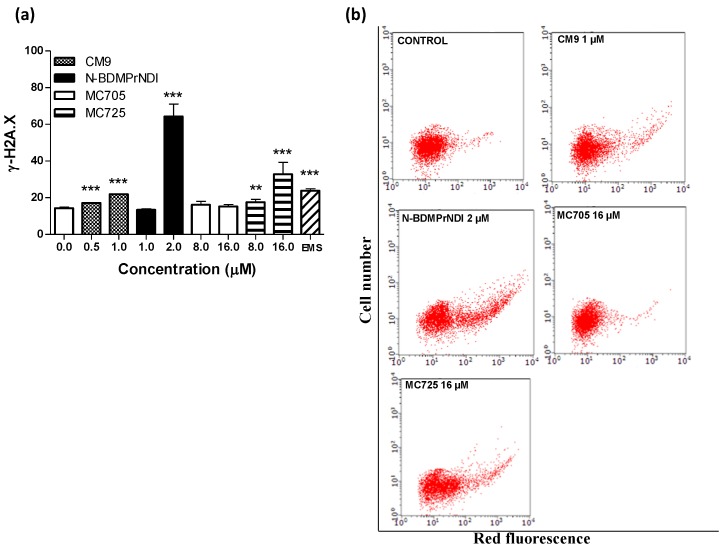
(**a**) Phosphorylation of histone γ-H2A.X induced by CM9, N-BDMPrNDI, MC705 or MC725 in Jurkat cells after 24 h treatment. Cells were treated with antibody against γ-H2A.X, and protein expression was determined by flow cytometry. EMS: ethyl methanesulfonate. ** *p* < 0.01, *** *p* < 0.001 *vs.* control; (**b**) Representative dot plots of Jurkat cultures untreated and treated with CM9, N-BDMPrNDI, MC725 or MC705 at the indicated concentrations for 24 h.

To better understand the pharmaco-toxicological potential of the tested compounds and contribute to the prediction of their risk/benefit profile, we analyzed their genotoxicity in Jurkat cells. The evaluation of H2A.X phosphorylation (γ-H2A.X) showed that CM9, N-BDMPrNDI and MC725 are genotoxic (Figure 8a,b). They actually induce a 1.5-fold, 4.5-fold and 2.5-fold increase of γ-H2A.X, respectively, at the highest tested concentrations compared to untreated cells (Figure 8a). On the other hand, MC725 was unable to significantly induce histone phosphorylation, therefore it cannot cause DNA double strands breaks. The genotoxicity of CM9 and N-BDMPrNDI is not surprising. Indeed, chemical compounds characterized by a NDI scaffold are able to intercalate and alkylate DNA structure [7,8,9,10,12]. Furthermore, different studies reported the genotoxicity of natural ITCs such as sulforaphane and allyl ITC [32,33]. On the whole, the above reported results indicate that the conjugation with fullerene does not reduce the reactivity of CM9 and N-BDMPrNDI with DNA. Since one of the most recognized apoptosis pathways runs through DNA damage, our results suggest that the induction of apoptosis recorded for CM9, N-BDMPrNDI and MC725 can be imputable to their genotoxic activity. Accordingly, the inability of MC705 to induce DNA damage can explain the lack of apoptotic effects reported above for this compound. However, the genotoxic potency of CM9 and MC725 is significantly lower than that of N-BDMPrNDI. This means that mechanisms other than induction of DNA damage may account for the proapoptotic potential of CM9 and MC725.

## 3. Experimental Section

### 3.1. Preparation of N-BDMPrNDI, CM9, MC705 and MC725

**Scheme 1:**
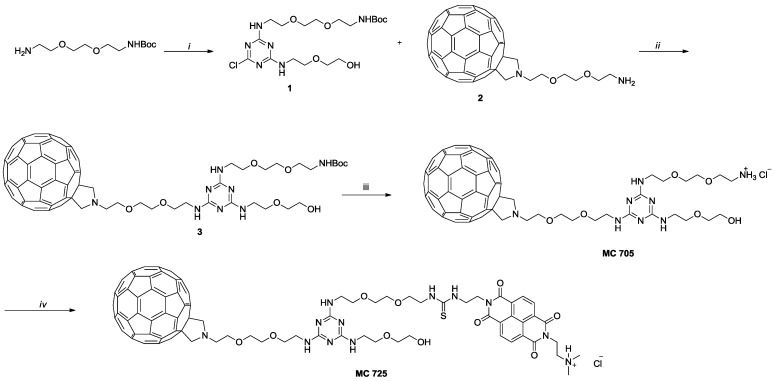
(*i*) a. cyanuric chloride, *N*,*N*-diisopropylethylamine (DIPEA), tetrahydrofuran (THF), 0 °C, 3 h; b. 2-(2-aminoethoxy)ethanol, DIPEA, THF, 0 °C to room temperature (rt), 18 h; (***ii***) diisopropyl ethyl amine (DIEA), orthodichlorobenzene (o-DCB), Ar, 80 °C, 40 h; (***iii***) HCl conc, dioxane, rt, overnight; (***iv***) CM9, DIEA, o-DCB, Ar, rt, overnight.

N-BDMPrNDI and CM9 have been synthesized following the procedure reported in literature [34]. Compounds MC705 and MC725 have been synthesized following the procedure depicted in Scheme 1. Briefly, chlorotriazine **1** has been obtained by reacting mono-boc protected 2,2'-(ethylenedioxy)*bis*(ethylamine), 2-(2-aminoethoxy)ethanol and cyanuric chloride [35]. **1** has been subject to nucleophilic aromatic substitution with **2** [36] to obtain compound **3** which was subjected to acidic hydrolysis leading to compound MC705. In the last step, the amino group of MC705 reacts with the ITC of CM9 leading to compound MC725 characterized by the thiourea moiety.

ESI-MS spectra were recorded on Perkin Elmer 297 (Perkin Elmer, Waltham, MA, USA) and Waters ZQ 4000 (Waters, Milford, MA, USA). ^1^H NMR and ^13^C NMR were recorded on Varian VRX 200 and 400 instruments (Varian, Palo Alto, CA, USA). Chemical shifts are reported in ppm relative to peak of tetramethylsilane. Infrared spectra were recorded on a Jasco FTIR-200 spectrometer (Jasco, Easton, MD, USA). Chromatographic separations were performed on silica gel columns by flash (Kieselgel 40, 0.040–0.063 mm, Merck, Darmstadt, Germany) column chromatography. Reactions were followed by thin layer chromatography (TLC) on Merck (0.25 mm) glass-packed precoated silica gel plates (60 F254) (Merck, Darmstadt, Germany) and then visualized in an iodine chamber or with a UV lamp VL-6.LC (Vilber Lourmat, Eberhardzell, Germany). The term “dried” refers to the use of anhydrous sodium sulfate.

*tert*-butyl(2-(2-(2-((4-chloro-6-((2-(2-hydroxyethoxy)ethyl)amino)-1,3,5-triazin-2-yl)amino)ethoxy)ethoxy)ethyl)carbamate (1). An ice-cooled solution of 269 mg of mono-boc protected 2,2'-(ethylenedioxy)*bis*(ethylamine) (1.48 mmol) and 566 μL of DIEA (420 mg, 3.253 mmol) in 20 mL of THF was added dropwise to an ice-cooled solution of 200 mg of cyanuric chloride (1.048 mmol) in 20 mL of THF. The mixture was stirred at 0 °C for 3 h. To this solution was added dropwise an ice-cold solution of 114 mg of 2-(2-aminoethoxy)ethanol (1.048 mmol) and 566 μL of DIEA (420 mg, 3.253 mmol) in 20 mL of THF. The resulting mixture was allowed to warm to rt and was stirred overnight. The solvent was evaporated and the residue was dissolved in dichloromethane (DCM) and washed with brine, dried over anhydrous Na_2_SO_4_ and purified by flash chromatography with EtOAc. 360 mg of **1** were obtained as white waxy solid (71% yield). ^1^H-NMR (200 MHz, CDCl3, 25 °C): δ 3.73 (br 2H), 3.62 (br 16H), 3.33 (br 2H), 1.43 (s 9H).

**(3).** 150 mg of Fullerene **2** (0.149 mmol) and 138 mg of **1** (0.297 mmol) were suspended in 26 mL of *o*-DCB, the mixture was degassed by means of 2 vacuum/Argon cycles. 4.20 mL of a solution of DIEA in *o*-DCB 10 mg/mL (42 mg, 0.327 mmol) were added. The resulting solution was stirred under Argon at 80 °C for 40 h. The reaction mixture was directly loaded on a flash chromatography column and eluted in gradient from Tol to Tol/EtOH 93:7. The product was precipitated from DCM/Et_2_O, washed with acetone/Et_2_O and finally with Et_2_O affording 128 mg of **3** as brown powder (65% yield). ^1^H-NMR (200 MHz, CDCl_3_, 25°C): δ 4.52 (s, 4H), 4.07 (t, *J* = 4.9 Hz, 2H), 3.92–3.45 (m, 26H), 3.78 (m, 4H), 1.44 (s, 9H). ^13^C-NMR (50 MHz, CDCl3, 25 °C): δ 171.52, 165.80, 155.26 (4C), 147.43 (2C), 146.37 (4C), 146.21 (4C), 146.19 (4C), 145.84 (2C), 145.52 (4C), 145.42 (4C), 144.69 (4C), 143.21 (2C), 142.74 (4C), 142.37 (4C), 142.21 (4C), 142.01 (4C), 140.27 (4C), 136.35 (4C), 79.41, 72.72, 71.07, 70.70, 70.45, 68.72, 61.92, 54.45, 40.73, 28.74. IR (NaCl): cm-1 3354, 2954, 2917, 2850, 1735, 1713, 1566, 1518, 1464, 1264, 1117, 737. ES-MS (MeOH) *m*/*z* = 1323.7 (MH^+^), 1345.6 (M + Na^+^).

**(MC705).** 85 mg of **3** (0.064 mmol) were suspended in 10 mL of dioxane/conc HCl 2:1 and the mixture was stirred overnight at rt The solvent was evaporated and the residue was precipitated from MeOH/Et_2_O and washed twice with Et_2_O, affording 76 mg of **MC 705** as brown powder (94% yield). ^1^H-NMR (200 MHz, DMSO-d6, 25°C): δ 8.36 (br, 1H, NH^+^), 7.99 (br, 3H, NH_3_^+^), 5.27 (br, 2H, NH) 4.19 (br, 2H), 4.74–3.36 (m, 28H), 2.95 (m, 2H). IR (ATR): cm-1 3255, 2872, 1630, 1563, 1450, 1323, 1120, 769. ES-MS (MeOH) *m*/*z* = 1223.6 (MH^+^), 612.5 (MH_2_^2+^).

**(MC725).** 25.42 mg of **MC705** (0.0202 mmol) were dissolved in 1.3 mL of dry MeOH under Argon. To this solution 1.3 mL of a solution of DIEA in *o*-DCB 10 mg/mL (13.04 mg, 0.1009 mmol) and 10.00 mg of **CM9** (0.0168) were added. The mixture was stirred at room temperature overnight, MeOH was evaporated and the product was purified by flash chromatography with using as eluent a mixture of CHCl_3_/MeOH 90:10. The product was precipitated from DCM/Et_2_O, washed with Et_2_O and re-precipitated as hydrochloride salt from DCM to DCM/Et_2_O saturated with HCl, washed with Tol and with Et_2_O. 20.54 mg of **MC725** were obtained as a brown powder (60% yield). ^1^H-NMR (200 MHz, CDCl_3_, 25°C, free base): δ 8.72 (s, 4H), 4.52 (s, 4H), 4.33 (m, 4H), 4.07 (t, *J* = 4.9 Hz 4H), 3.80 (m, 30H), 3.54 (m, 8H), 2.69 (t, *J* = 6.4 Hz, 1H), 2.35 (s, 6H). IR (ATR): cm-1 3229, 2944, 2869, 1703, 1664, 1628, 1558, 1454, 1337, 1248, 1114, 768. ES-MS (MeOH) *m*/*z* = 1646.4 (MH^+^), 1681.2 (M + Cl^−^).

### 3.2. Cell Cultures

Human Jurkat T leukemia cells were purchased from Istituto Zooprofilattico of Brescia (Brescia, Italy). Human Raji lymphoma cells were kindly provided by Prof. Maurizio Brigotti (Dipartimento di Medicina Specialistica, Diagnostica e Sperimentale, Università di Bologna, Bologna, Italy). Cells were grown in suspension and propagated in RPMI 1640 supplemented with 10% heat-inactivated bovine serum, 1% penicillin/streptomycin solution and 1% L-glutamine solution (all obtained from Sigma Aldrich, Saint Louis, MO, USA). Cells were incubated at 37 °C with 5% CO_2_. To maintain exponential growth, the cultures were divided every third day by dilution to a density of 1 × 10^5^ cells/mL.

### 3.3. Cell Treatment

Cells were treated with increasing concentrations of CM9 (0.0–4.0 μM), *N*-BDMPrNDI (0.0–4.0 μM), MC705 (0.0–32.0 μM) or MC725 (0.0–32.0 μM) for 24 h (*i.e.*, one cell cycle). Cells were also treated with CM9 (0.5, 1.0 μM) plus (0.25, 0.50 μM). Camptothecin (Sigma Aldrich, St. Louis, MO, USA) 0.25 μM was used as positive control.

### 3.4. Cell Viability and Detection of Apoptosis

Cells viability was determined using Guava ViaCount Reagent (Merck Millipore, Darmstadt, Germany), containing 7-amino-actinomycin D (7-AAD). Briefly, cells were appropriately diluted with the reagent and incubated at room temperature in the dark for 5 min before detection with flow cytometer. Additionally, Guava Nexin Reagent (Merck Millipore, Darmstadt, Germany), containing 7-AAD and annexin V-phycoerythrin (annexin V-PE), was used to discriminate between apoptotic and necrotic cells. According to manufacturer’s instructions, cells were incubated with the reagent for 20 min at room temperature in the dark and then analyzed via flow cytometry. IC_50_, the inhibitory concentration causing cell toxicity by 50% following one cell-cycle exposure, was calculated. Concentrations ≤ IC_50_ were used in the subsequent experiments.

### 3.5. Cell-Cycle Analysis

Cells were treated with MC705 or MC725 for 24 h and then fixed with 70% ice-cold ethanol. Samples were washed, suspended in 200 μL of Guava Cell Cycle Reagent (Merck Millipore, Darmstadt, Germany), containing propidium iodide, and incubated at room temperature for 30 min in the dark before flow cytometric analysis.

### 3.6. DNA Damage

To analyze the genotoxic potential of the tested compounds, the phosphorylation of histone γ-H2A.X was evaluated, as marker of DNA double strand breaks. Briefly, after 24 h of treatment, cells were fixed, permeabilized and incubated for 30 min in the dark at room temperature with an anti γ-H2A.X-Alexa Fluor^®^ (Merck Millipore, Darmstadt, Germany). Ethyl methanesulfonate (EMS, 240 μg/mL, Sigma Aldrich, St. Louis, MO, USA) was used as positive control. Cells were analyzed *via* flow cytometry.

### 3.7. Detection of Caspase-3 Activity

Caspase-3 activity was evaluated after 24 h treatment with CM9. Briefly, 10 μL of FAM-DEVD-FMK 10X solution (Merck Millipore, Darmstadt, Germany) was added to cell suspension and then incubated for 1 h at 37 °C and 5% CO_2_. After washing, cells were suspended in 150 μL of 7-AAD (Merck Millipore, Darmstadt, Germany) for 5 min at room temperature in the dark and analyzed via flow cytometry.

### 3.8. Analysis of p53, PARP, Bax, Bcl-2, and Cyclin E Proteins

After CM9 treatment for 24 h, aliquots of 1 × 10^6^ cells were fixed with a 4% formaldehyde solution and permeabilized with 90% cold methanol solution. Cells were mixed with the specific primary antibodies: FITC-anti-p53 (20 μL, BD Biosciences, San Jose, CA, USA), FITC-anti-PARP (1:25, Invitrogen, Carlsbad, CA, USA), antibody specific for the 85 kDa PARP cleaved portion, anti-Bax (1:100, Santa Cruz Biotechnology, Santa Cruz, CA, USA), anti Bcl-2 (1:100, Santa Cruz Biotechnology, Santa Cruz, CA, USA), anti-cyclin E (1:10, Santa Cruz Biotechnology, Santa Cruz, CA, USA), or an adequate volume of isotype-matched negative control (1:500, e-Bioscience, San Diego, CA, USA). Cells were washed and incubated with the FITC-conjugated secondary antibody. Cells, labeled with FITC-conjugated isotype control, were gated to exclude non-specific binding.

### 3.9. Flow Cytometry

EasyCyte 5HT (Merck Millipore, Darmstadt, Germany) was used to perform all flow cytometric analyses. For each sample, approximately 5000 events were evaluated.

### 3.10. Statistical Analysis 

All results are expressed as mean ± SEM of at least three experiments. Differences between treatments were assessed by t test or one way ANOVA and Dunnet or Bonferroni was used as post-tests. All statistical analyses were performed using GraphPad InStat 5.0 version (GraphPad Prism, San Diego, CA, USA). *p* < 0.05 was considered significant. IC_50_ values was calculated using Probit [37]. To determine the effect of the combination CM9 plus doxorubicin, the combination index (CI) was used. CI analysis provides qualitative information on the nature of drug interaction, and a quantitative measure of the extent of the interaction [38].

CI=[A]x[IA]x+[B]x[IB]x

[A]*_x_* and [B]*_x_* are the concentrations of drugs A and B used in combination to obtain *X*% of drug effect. [IA]*_x_* and [IB]*_x_* are the concentrations of individual agents to achieve the same effect. CI = 1 indicates additive effect, CI < 1 synergistic effect, CI > 1 antagonism.

## 4. Conclusions

Our results together show that the synthesized ITC CM9 reduced significantly the viability of Jurkat leukemia cells in a concentration-dependent manner. The mechanism of cell death induced by CM9 involves the modulation of Bcl-2 and Bax expression and increase in active caspase-3, activating the intrinsic pathway and execution phase of apoptosis. Thus, CM9 is a bioactive compound in leukemia cells and could represent a promising prototype of drugs for acute leukemia treatment. Notably in this context, the concentration required to reach IC_50_ for two well-known natural ITCs like sulforaphane and phenethyl isothiocyanate in the same cell line was about 8-fold and 4-fold higher, respectively, than that detected for CM9 [1,39]. Furthermore, phenethyl isothiocyanate induced a marked increase in activation of caspase-3 as well as PARP degradation only at concentrations ≥6 μM [40].

The conjugation of CM9 with a non-toxic vector, e.g., MC705, allowed the obtention of a new derivative (MC725), through the formation of a thiourea. Thiourea is stable in cells, thus preventing the release of CM9. Accordingly, the anticancer potential of CM9 is only partly maintained and the conjugation does not increase its anticancer potential. This opens an interesting research pattern on fullerene properties. The conjugation of MC725 with a fluorescent marker allows the definition of its intracellular concentration and therefore the understanding as to whether the presence of fullerene increases the delivery and the intracellular concentration of CM9. Furthermore, future experiments could be addressed to investigate the cytotoxic and cytostatic activity of a mixture of fullerene plus CM9. Effectively, it has been shown that a mixture of metallofullerene nanoparticles plus anticancer drugs can favor accumulation of intracellular chemotherapeutic drugs such as cisplatin [41]. In this strategy, CM9 would keep free its ITC group. Accordingly, treatment of cells with a mixture and not a conjugate of fullerene with CM9 might facilitate the entry of CM9 in cells, where its free ITC group might react with different molecular targets, as reported for natural ITCs [1]. Finally, the introduction of a target delivery molecule on the fullerene carbon cage or on the free amine terminating chain might increase the selectivity of the construct.
